# Multicentre comparison of quantitative PCR-based assays to detect SARS-CoV-2, Germany, March 2020

**DOI:** 10.2807/1560-7917.ES.2020.25.24.2001057

**Published:** 2020-06-18

**Authors:** Maximilian Muenchhoff, Helga Mairhofer, Hans Nitschko, Natascha Grzimek-Koschewa, Dieter Hoffmann, Annemarie Berger, Holger Rabenau, Marek Widera, Nikolaus Ackermann, Regina Konrad, Sabine Zange, Alexander Graf, Stefan Krebs, Helmut Blum, Andreas Sing, Bernhard Liebl, Roman Wölfel, Sandra Ciesek, Christian Drosten, Ulrike Protzer, Stephan Boehm, Oliver T Keppler

**Affiliations:** 1 Max von Pettenkofer Institute and Gene Center, Virology, National Reference Center for Retroviruses, Ludwig Maximilian University, Munich, Germany; 2 German Center for Infection Research, Partner Site Munich and Associated Partner Site Charité, Berlin and Associated Partner Site Frankfurt, Germany; 3 Institute of Virology, School of Medicine, Technical University Munich/Helmholtz Zentrum München, Munich, Germany; 4 Institute of Medical Virology, University Hospital, Goethe University Frankfurt am Main, Frankfurt, Germany; 5 Bavarian Health and Food Safety Authority, Oberschleißheim, Germany; 6 Bundeswehr Institute of Microbiology, Munich, Germany; 7 Laboratory for Functional Genome Analysis (LAFUGA), Gene Center, Ludwig Maximilian University, Munich, Germany; 8 Institute of Virology, Charité University Medicine, Berlin, Germany

**Keywords:** SARS-CoV-2, COVID-19, PCR, diagnostic test

## Abstract

Containment strategies and clinical management of coronavirus disease (COVID-19) patients during the current pandemic depend on reliable diagnostic PCR assays for the severe acute respiratory syndrome coronavirus 2 (SARS-CoV-2). Here, we compare 11 different RT-PCR test systems used in seven diagnostic laboratories in Germany in March 2020. While most assays performed well, we identified detection problems in a commonly used assay that may have resulted in false-negative test results during the first weeks of the pandemic.

Strategies to limit the severe pandemic and to manage coronavirus disease (COVID-19) patients strongly depend on readily available, accurate and reliable RT-PCR assays to detect the genome of the causative agent acute respiratory syndrome coronavirus 2 (SARS-CoV-2) in biosamples. The first full-length SARS-CoV-2 genome sequence was made publicly available in early January 2020 [1] and, soon after, various RT-PCR assays were reported by academic laboratories, public health agencies and diagnostics companies [2-6]. Their overall performance and relative sensitivity are largely unclear.

The aim of this study was to compare the inter-laboratory and inter-method sensitivity of different RT-PCR assays by providing a blinded, frozen dilution series of a nucleic acid extract of a highly positive biosample to seven different diagnostic laboratories in Germany in March 2020.

## Sample preparation and study design

Nucleic acids were pooled from multiple extractions of one SARS-CoV-2-positive stool sample using the QIAsymphony DSP Virus/Pathogen Kit (Qiagen, Hilden, Germany). This stool sample was from a 5-year-old child with COVID-19 [7] and was chosen because of high initial PCR signals and sufficient sample availability to generate large quantities of eluate for further distribution. Of note, no PCR inhibition was observed for detection of the spiked-in extraction RNA control (QuantiNova IC Probe Assays Red 650, Qiagen). A 1:10 dilution series was prepared and aliquots were labelled in a blinded fashion to be shipped on dry ice to participating laboratories in March 2020. Participants were instructed to perform the diagnostic assays used at their centre for SARS-CoV-2 detection in quadruplicate using 5 µL of the aliquot per reaction. All results were reported back to the initiating laboratory (Laboratory 1) before the results were unblinded. The details of all these PCR-based assays are summarised in the Table.

**Table:** Specifications of different molecular assays used for detection of SARS-CoV-2, Germany, March 2020 (n =11 test systems with 34 different reaction–lab combinations)

Laboratory	Protocol	Target	Primer/probe	Supermix	Instrument
Laboratory 1	CDC [2]	N1, N2, N3	Ella Biotech	QuantiNova Multiplex RT-PCR Kit	Roche LightCycler 480 II
	Charité [3,4]	E, N, RdRp	Tib-Molbiol	QuantiNova Multiplex RT-PCR Kit	Roche LightCycler 480 II
	Modified Charité RdRp primers	RdRp	Ella Biotech	QuantiNova Multiplex RT-PCR Kit	Roche LightCycler 480 II
	Applied Biosystems TaqMan 2019-nCoV Assay Kit v1	S, N, RdRp	Commercial kit	TaqMan Fast Virus 1-Step Master Mix	Applied Biosystems 7500 fast
	Seegene Allplex 2019-nCoV Assay	E, N, RdRp	Commercial kit	Commercial kit	Biorad CFX 96 Real-Time System
	Digital droplet PCR using CDC primer and probe sequences	N1, N2, N3	Ella primers/IDT ZEN Double-Quenched Probe	BioRad 1-Step RT-ddPCR Advanced Kit for Probes	Biorad QX200 droplet digital PCR
	Digital droplet PCR using Charité primer and probe sequences	E, N, RdRp	Ella primers/IDT ZEN Double-Quenched Probe	BioRad 1-Step RT-ddPCR Advanced Kit for Probes	Biorad QX200 droplet digital PCR
Laboratory 2	Charité [3,^4^]	E, RdRp	Tib-Molbiol	Superscript III One-Step RT-PCR System With Platinum Taq Polymerase	Roche LightCcycler 480 II
Laboratory 3	Charité [3,4]	E, RdRp (2 or 1 and 2)	Tib-Molbiol	Superscript III One-Step RT-PCR System With Platinum Taq Polymerase	Biorad CFX 96 Real-Time System
	Altona diagnostics RealSstar SARS-CoV-2 RT-PCR	Beta-CoV, SARS-CoV-2	Commercial kit	Commercial kit	Biorad CFX 96 Real-Time System
Laboratory 4	Charité [3,4]	E, RdRp	Tib-Molbiol	RNA to CT 1-step	Applied Biosystems 7500 fast
	Laboratory developed test	M, S	Tib-Molbiol	Roche Multiplex RNA Virusmaster	Roche LightCycler 480 II
Laboratory 5	Charité [3,4]	E, N, RdRp	Tib-Molbiol	Quantitect Virus +ROX Vial Kit	Applied Biosystems 7500 fast
	CDC [2]	N1, N2, N3	Microsynth	Quantitect Virus +ROX Vial Kit	Applied Biosystems 7500 fast
Laboratory 6	Charité [3,4]	E, N, RdRp	Tib-Molbiol	Qiagen one step RT-PCR Kit	Bio Molecular Systems MIC Cycler
Laboratory 7	Mikrogen ampliCube Coronavirus Panel	Various coronaviruses	Commercial kit	Commercial kit	Roche LightCycler 480 II
	Mikrogen ampliCube Coronavirus SARS-CoV-2	E, Orf1a	Commercial kit	Commercial kit	Roche LightCycler 480 II

Beta-CoV: Betacoronavirus; CDC: Centers for Disease Control and Prevention; E: envelope gene; N: nucleocapsid gene; Orf: open reading frame; RdRp: RNA-dependent RNA-polymerase gene; SARS-CoV-2: severe acute respiratory syndrome coronavirus 2; WHO: World Health Organization.

Performing laboratory, assay protocol, target, manufacturer of primer/probe, PCR chemistry and instrument are indicated.

**Figure 1:**
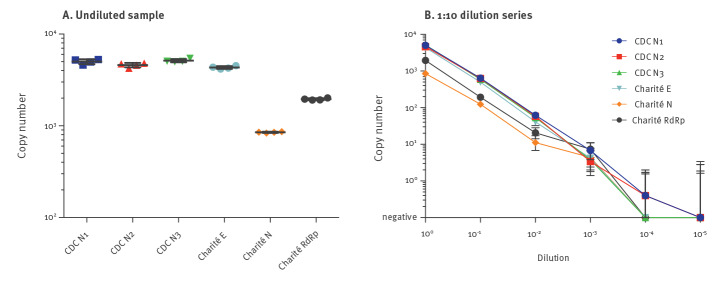
Digital droplet PCR quantification of the distributed dilution series of nucleic acid eluate of SARS-CoV-2-positive clinical material, Germany, March 2020

CDC: Centers for Disease Control and Prevention; E: envelope gene; N: nucleocapsid gene; RdRp: RNA-dependent RNA-polymerase gene; SARS-CoV-2: severe acute respiratory syndrome coronavirus 2.

In parallel, samples were quantified using the One-Step RT-digital droplet (dd)PCR Advanced Kit for Probes (BioRad, Feldkirchen, Germany) on the BioRad QX200 platform. Primer and probe sequences were used for detection of the SARS-CoV-2 *nucleocapsid* gene (*N*) as published by the Centers for Disease Control and Prevention (CDC) [2] and the ﻿*envelope* gene (*E*), the ﻿*RNA-dependent RNA polymerase* (*RdRp*) gene and the *N* gene as published by Corman et al. (referred to as Charité protocol) [3] (Figure 1).

The RNA copy numbers are indicated for each PCR reaction using either the CDC and the Charité primer/probe combinations and were measured from 5 µL nucleic acid eluate. Mean values of quadruplicates are indicated by the horizontal lines (A) or symbols (B). Error bars represent the 95% Poisson confidence interval (B).

The undiluted sample showed between 4,325 and 5,015 SARS-CoV-2 RNA copies per reaction using 5 µL of eluate for the CDC *N1*, *N2*, *N3* and Charité *E *protocols, but only 850 and 1,951 RNA copies for the Charité *N* and *P* primer/probe combinations (Figure 1A), respectively, indicating a lower sensitivity of the latter. The 1:10 dilution series displayed good linearity down to a calculated concentration of 0.4 RNA copies per reaction at the 10^−4^ dilution for both the CDC *N1* and *N2* primer/probe combinations (Figure 1B).

## Multicentre and multi-assay comparison

**Figure 2:**
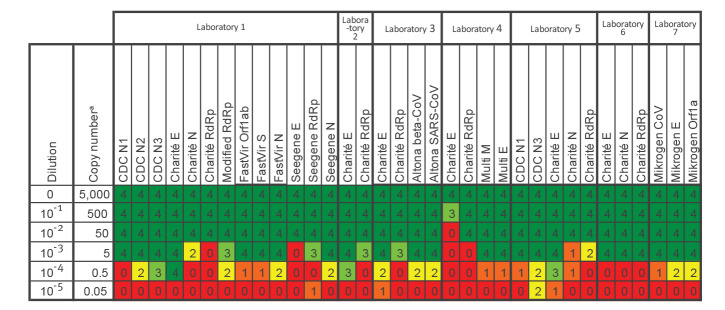
Dilution series comparing various RT-PCR assays for the detection of SARS-CoV-2 at different laboratories, Germany, March 2020 (n =11 test systems with 34 reaction–lab combinations)

Beta-CoV: Betacoronavirus; CDC: Centers for Disease Control and Prevention; E: envelope gene; N: nucleocapsid gene; ORF: open reading frame; RdRp: RNA-dependent RNA-polymerase gene; S: spike gene; SARS-CoV-2: severe acute respiratory syndrome coronavirus 2.

^a^ Copy numbers are estimated based on ddPCR results shown in Figure 1.

Result interpretations from the seven participating laboratories are summarised in Figure 2 displaying the number of replicates scored positive by the respective laboratory for each method and dilution. Most methods reliably detected the sample at the 10^−3^ dilution, which is equivalent to ca 5 RNA copies for the CDC *N1*, *N2*, *N3* and Charité *E* reactions based on the absolute quantification by ddPCR. Of note, the Seegene Allplex 2019-nCoV Assay gave negative results for all four replicates in the *E* gene at the 10^−3^ dilution, while reporting positive results for *N* and *RdRp* (Laboratory 1). According to the manufacturer’s instructions at the time of analysis, this would have been interpreted as an inconclusive result. Of note, the *RdRp* primer/probe did not show any positive result at the 10^−4^ dilution.

A 1:10 dilution series was prepared from pooled eluates of one SARS-CoV-2 stool sample and sent to participating laboratories to be tested in quadruplicate. The number of positive replicates is indicated and colour-coded as a heatmap with four positive results shown in green and four negative results shown in red.

## Sequence analysis of primer pairs

Driven by false-negative results for samples with low PCR-positivity using the original Charité *RdRp* reaction (see below and others [8,9]), we compared the primer/probe sequences with currently available SARS-CoV-2 genomes. When compared with all genomes available on GISAID (9,184 SARS-CoV-2 genomes on 15 April 2020, Supplement), the regions used for amplification in the CDC and Charité protocol are highly conserved: Only 1.55%, 0.45% and 2.4% of genome sequences contain any kind of mismatch within the primer/probe regions of the CDC *N1*, *N2* and *N3* protocols, respectively, and 0.25%, 0.29% and 0.67% in the primer/probe regions of the Charité *E*, *RdRp* and *N* protocols, respectively.

The Charité *RdRp* reverse primer contains an ambiguity base at position 15,519 that does not match the reference sequence (Wuhan-Hu-1/2019), with an S (i.e. G or C) instead of T for the reverse complement (Supplementary Figure S1). The other ambiguity base at 15,528 showing Y (i.e. C or T) should be changed to T because the currently circulating viruses have a T at this position and no polymorphisms were detected in any of the 9,184 sequences submitted to date (accession date: 15 April 2020). Based on computation using Primer Express v3.0 (Applied Biosystems, Dreieich, Germany) annealing temperatures were predicted to be 64 °C for the *RdRp* forward and 51 °C for the *RdRp* reverse primer of the Charité protocol. This temperature difference may result in reduced PCR efficiency. To address this issue, modified *RdRp* primers were synthesised as shown in Supplementary Figure S1 and tested in comparison with the original primers. 

## Differential detection of respiratory samples with low PCR positivity 

**Figure 3:**
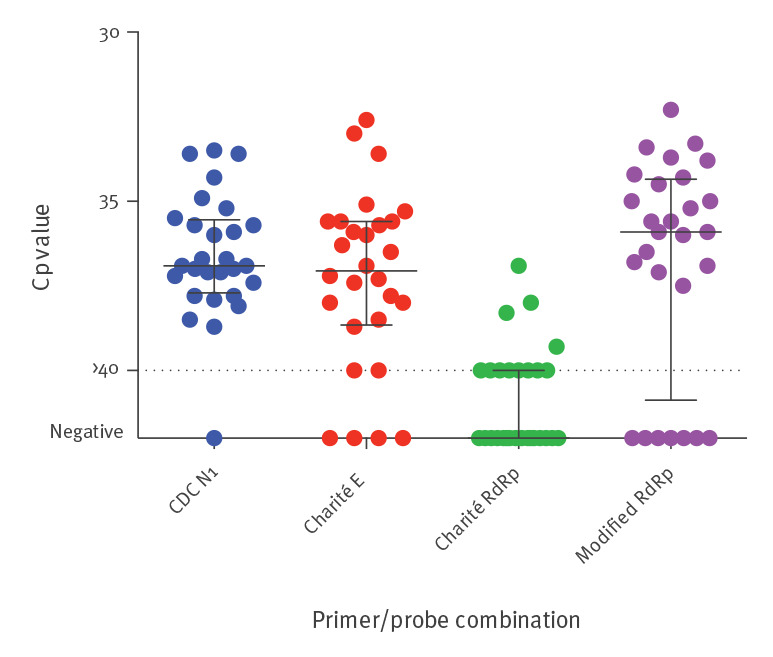
RT-PCR results of respiratory samples with low positivity, SARS-CoV-2 detection, Germany, March 2020 (n = 28 samples)

CDC: Centers for Disease Control and Prevention; Cp: crossing point; E: envelope gene; N: nucleocapsid gene; RdRp: RNA-dependent RNA-polymerase gene; SARS-CoV-2: severe acute respiratory syndrome coronavirus 2.

Testing the dilution series with these modified *RdRp* primers (see above and Supplementary Figure S1) yielded positive results for two additional dilution steps (10^-3^ and 10^-4^) compared with the original Charité *RdRp* primers (Figure 1). To further compare the sensitivity of these modified *RdRp* primers with the original version of the Charité *RdRp* primers and the Charité *E* and the CDC *N1* reaction, we retested 28 eluates of clinical respiratory specimens from the diagnostic unit at Laboratory 1 that had shown crossing point (Cp) values > 35 in the initial CDC *N1* reaction. Using the original version of the confirmatory Charité *RdRp* primers, 16 of 28 samples tested negative, but 11 of these showed positive results using the modified primers (Figure 3). Overall, the detection by the Charité *E*, modified Charité *RdRp*, and CDC *N1* reactions were robust. Notably, six and seven of these 28 respiratory samples scored negative or at the limit of detection (Cp = 40) in the Charité *E* and modified Charité *RdRp* reactions, while only one sample came up negative in retesting in the CDC *N1* reaction (p = 0.04 and p = 0.02, chi-squared-test comparing Charité *E* and modified *RdRp* to CDC *N1*, respectively). Of note, in a routine clinical setting, the CDC *N1* reaction also detected SARS-CoV-2 RNA in nucleic acid extracts from 37 of 83 sera (45%) from COVID-19 patients in intensive care units, with a positive correlation of their Cp values with those of the corresponding respiratory material (Spearman Rank correlation co-efficient r=0.4285, p (two-tailed) < 0.0001 (data not shown)).

Nucleic acid eluates of clinical respiratory specimens that initially showed low positive results in the CDC *N1* reaction (Cp value > 35) were retested side by side in the CDC *N1*, the Charité *E* and original *RdRp* reaction and using the modified *RdRp* primers on the Roche LightCycler 480 using the QuantiNova Multiplex RT-PCR kit. Cp values are shown with positive amplifications beyond cycle 40 shown as > 40 (dotted line).

## Conclusion

The majority of RT-PCR assays for SARS-CoV-2 examined in this study detected ca 5 RNA copies per reaction, reflecting a high sensitivity and their suitability for screening purposes world-wide. A reduced sensitivity was noted for the original Charité *RdRp* gene confirmatory protocol, which may have impacted the confirmation of some COVID-19 cases in the early weeks of the pandemic. The protocol needs to be amended to improve the sensitivity of the *RdRp* reaction. The CDC *N1* primer/probe set was sensitive and robust for detection of SARS-CoV-2 in nucleic acid extracts from respiratory material, stool and serum from COVID-19 patients.

## Supplementary Data

46000 Supplement

## Supplementary Data

355000 Supplementary Figure
