# Supplementary material for: Multicentre comparison of quantitative PCR-based assays to detect SARS-CoV-2, Germany, March 2020
Source: Euro Surveill. 2020 Jun 18;25(24):2001057. doi: 10.2807/1560-7917.ES.2020.25.24.2001057 (PMC7315722; doi:10.2807/1560-7917.ES.2020.25.24.2001057)
Supplement: Supplementary Figure [file 20-01057_KEPPLER_Supplementary_Figure_S1.pdf]

Supplementary Figure S1: Alignment of the original Charité RdRp forward and reverse primers with reference sequence and modified primers.

This supplementary material is hosted by Eurosurveillance as supporting information alongside the article "Multicentre comparison of quantitative PCR-based assays to detect SARS-CoV-2, Germany, March 2020", on behalf of the authors, who remain responsible for the accuracy and appropriateness of the content. The same standards for ethics, copyright, attributions and permissions as for the article apply. Supplements are not edited by Eurosurveillance and the journal is not responsible for the maintenance of any links or email addresses provided therein.

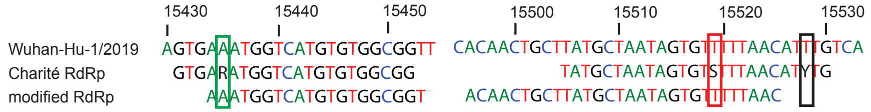

The forward primer and the reverse complement of the reverse primer of the RdRp reaction from the Charité protocol is aligned to the reference sequence Wuhan-Hu-1/2019 (NCBI NC_045512.2). The red box indicates an ambiguity base S, i.e. G or C, at a position where T should be the reverse complement. The black box indicates an ambiguity base Y, i.e. T or C, at a position where T would be sufficient, and the green box indicates an R where A can be used based on currently available sequence data.
